# Capturing SARS-CoV-2 immune landscapes to inform future strategies for COVID-19 vaccination in a high-income setting: a mathematical modelling study

**DOI:** 10.1186/s12879-026-13074-3

**Published:** 2026-03-25

**Authors:** Alexandra B. Hogan, David J. Muscatello, Bette Liu, Gemma Nedjati-Gilani, James G. Wood

**Affiliations:** 1 School of Population Health, Faculty of Medicine and Health, UNSW Sydney, Sydney, Australia; 2 National Centre for Immunisation Research and Surveillance, Sydney, Australia; 3 MRC Centre for Global Infectious Disease Analysis, Imperial College London, London, UK

**Keywords:** COVID-19 pandemic, Mathematical infectious disease model, COVID-19 vaccination, Scenario modelling, SARS-CoV-2

## Abstract

**Background:**

In an era of endemic SARS-CoV-2 transmission, countries are continuing to evaluate how best to schedule ongoing COVID-19 booster vaccinations. Mathematical modelling provides a useful tool to predict the benefit of future vaccination strategies, incorporating the loss of protection due to waning immunity and strain mutation.

**Methods:**

We adapted a combined immunological-population transmission model for SARS-CoV-2, to better capture contemporary understanding of exposure- and vaccine-derived immunity, to simulate ongoing endemic transmission of SARS-CoV-2 in a highly exposed high-income setting. We used this model to estimate the impact of targeted booster dose strategies in the older population, both in the context of continued circulation of the current dominant viral strain, and in the presence of a new antigenically distinct variant.

**Results:**

We found that at the population level, an annual COVID-19 vaccine booster dose to the 65+ years population at 60% coverage could avert 8–16% of hospitalisations over a single wave, depending on how well-matched the vaccine is to the circulating SARS-CoV-2 strain. With lower coverage of 40%, estimated median impact was between 6% and 11%. A second booster dose to the 75+ population after 6 months was particularly beneficial if a new distinct variant strain increases the magnitude of the wave. Of the vaccine scenarios explored, we found that increasing uptake of the annual booster dose in the 65+ population is likely to have a larger impact on hospitalisations than optimising dose timing.

**Conclusions:**

This adapted model captures endemic viral transmission and could readily be used to explore vaccine impact across other settings.

**Supplementary Information:**

The online version contains supplementary material available at 10.1186/s12879-026-13074-3.

## Background

On 5 May 2023, the World Health Organization (WHO) declared that COVID-19 would no longer be considered a public health emergency of international concern [1]. The SARS-CoV-2 virus is now characterised by endemic transmission in all countries, with periodic waves due to immunity waning following infection or vaccination, seasonality of infection, and immune escape as the virus evolves [2]. Despite the cessation of the emergency, COVID-19 remains an ongoing cause of respiratory morbidity and death globally [3]. In Australia, COVID-19 continues to cause hospitalisations, intensive care unit (ICU) admissions, deaths, and aged care outbreaks, and was the leading cause of acute respiratory infection mortality across 2022–2024 [4, 5]. The burden of COVID-19 hospitalisation and severe outcomes remains on average higher than that for influenza [6].

Most of the population globally has likely been exposed to SARS-CoV-2 multiple times over the past five years, and combined with vaccination, this has provided hybrid immunity against infection and disease [7–9]. However, protection from both vaccines and infection wanes over time [2]. Since the emergence of Omicron, the majority of immune boosting has occurred through exposure to the virus, and vaccination now remains primarily a tool for reducing severe COVID-19 disease and death. This is particularly true in older individuals, those with additional health conditions that increase their risk, and priority populations [10].

In September 2024, WHO’s Strategic Advisory Group on Immunization (SAGE) updated its COVID-19 booster dose advice, recommending 12-monthly booster doses in adults over 50 or 60 years, and 6-monthly boosters in adults over 75 or 80 years, with the age cut-off depending on country, and broader vaccination recommended in individuals with comorbidities and in other priority risk groups [11]. In Australia, booster doses are now recommended every 12 months for the 65+ years population, and every 6 months for the 75+ years population [12]. An additional consideration is vaccine composition; as the virus has evolved, vaccines have been updated to better target the most common circulating strains [13]. WHO SAGE recommends being vaccinated with whatever product is available at the time of immunisation. Even though advice on continuing annual or biannual booster doses remains in place, vaccine uptake is moderate. For example, by the end of November 2024, the United Kingdom Health Security Agency (UKHSA) reported that around 56% of 65+ year-olds had been vaccinated with an autumn 2024 booster dose [14], and according to recent Australian data, around 34% of 65–74-year-olds and around 50% of 75+ year-olds had received a COVID-19 booster dose in the last 12 months [15].

Countries now need to plan ongoing scheduling of booster vaccine doses in the context of waning immunity and continual emergence of new variants that evade existing immunity. Mathematical models of COVID-19 transmission and vaccination were important tools for informing health policy decisions throughout the pandemic [16]. However, now that we have moved beyond the emergency response era of COVID-19 control, there is a need to develop models that can inform vaccination planning over longer-term time horizons [17]. To do this, models will need to capture the transition to endemic SARS-COV-2 transmission in non-naïve populations with complex and varied vaccination and exposure histories, in the context of high prior infection exposure.

In our study we adapted an existing individual-based population-level transmission model of SARS-CoV-2 transmission and disease, linked to an individual-level immunity model, to capture the relative protection derived from virus exposure and vaccination. Using this model, we generated scenarios for endemic transmission, aiming to capture population-level immunity and levels of infection that would be broadly representative of Australia. We used this model to estimate the future impact of different targeted vaccination scenarios, considering the relative impact of how well-matched the vaccine is to the current circulating variant, the impact of booster dose timing, and the value of vaccination in the context of the emergence of new distinct variant strains.

## Methods

### Modelling approach

Our modelling approach is comprised of two steps. First, focusing on immunity within an individual person, we use an immunological model that simulates SARS-CoV-2 immune recognition over time [18]. Within the individual, immune recognition can be boosted by either vaccination or infection and is assumed to decay over time. We then apply a logistic function that captures the relationship between immune recognition and protection from both infection and severe disease [18]. Second, we simulate these processes across the population by implementing the individual-level immunological protection model within an existing framework of SARS-CoV-2 transmission and disease [19].

The modelling framework has been applied previously in the context of estimating the benefit of specific vaccine products, following the emergence of the Omicron variant, but is updated for the present analysis [18, 19]. Specifically, in our study, we update the combined immunological-population model [19] to capture endemic SARS-CoV-2 transmission in a population with high previous infection incidence and chequered vaccine histories. We do this by:

(i) reformulating infection- and vaccine-induced protection processes at the individual level, to better represent current understanding of protection and durability following exposure versus vaccination;
(ii) reconstructing the implementation of vaccine- versus infection-derived immunity within the population-level model; and.
(iii) recalibrating the model to capture characteristics of contemporary transmission patterns and recent effectiveness data.

The approach is detailed below.

### Individual-level immunological model

The dynamics of the immunological model are fully described elsewhere [18, 19] and are summarised as follows. Briefly, we follow the approach presented by Khoury et al. [20], in that an individual’s immunity following either infection or vaccination decays exponentially in a biphasic fashion, with a short period of fast decay followed by a longer period of slow decay. This immunity is related to efficacy against infection and severe disease by a logistic function (see Supplementary Information) [20]. Expressing the immunological model in this way allows an estimated fold-change reduction in immune recognition (for example by the emergence of a new variant) to be translated to reductions in efficacy against mild and severe disease. In our modelling approach, we conceptualise “immune recognition” to the SARS-CoV-2 virus antigen at the individual level, as a broad level of immunity (which can be comprised of a range of immune mechanisms, including B-cell and T-cell-mediated immunity [21, 22]), in response to the currently circulating SARS-CoV-2 variant.

An individual’s level of immune recognition can be boosted by either infection or vaccination. This immune recognition then wanes over time, according to the process described above. For each simulated individual, the infection-acquired and vaccine-acquired levels of immune recognition are allocated separately, and decay independently. The decay dynamics of infection- and vaccine-acquired immune recognition are similar, but we allow for different levels of boosting from infection and vaccination, and additionally account for the extent that a vaccine is matched to the circulating variant (either “well-matched” or “partially-matched”).

**Fig. 1:**
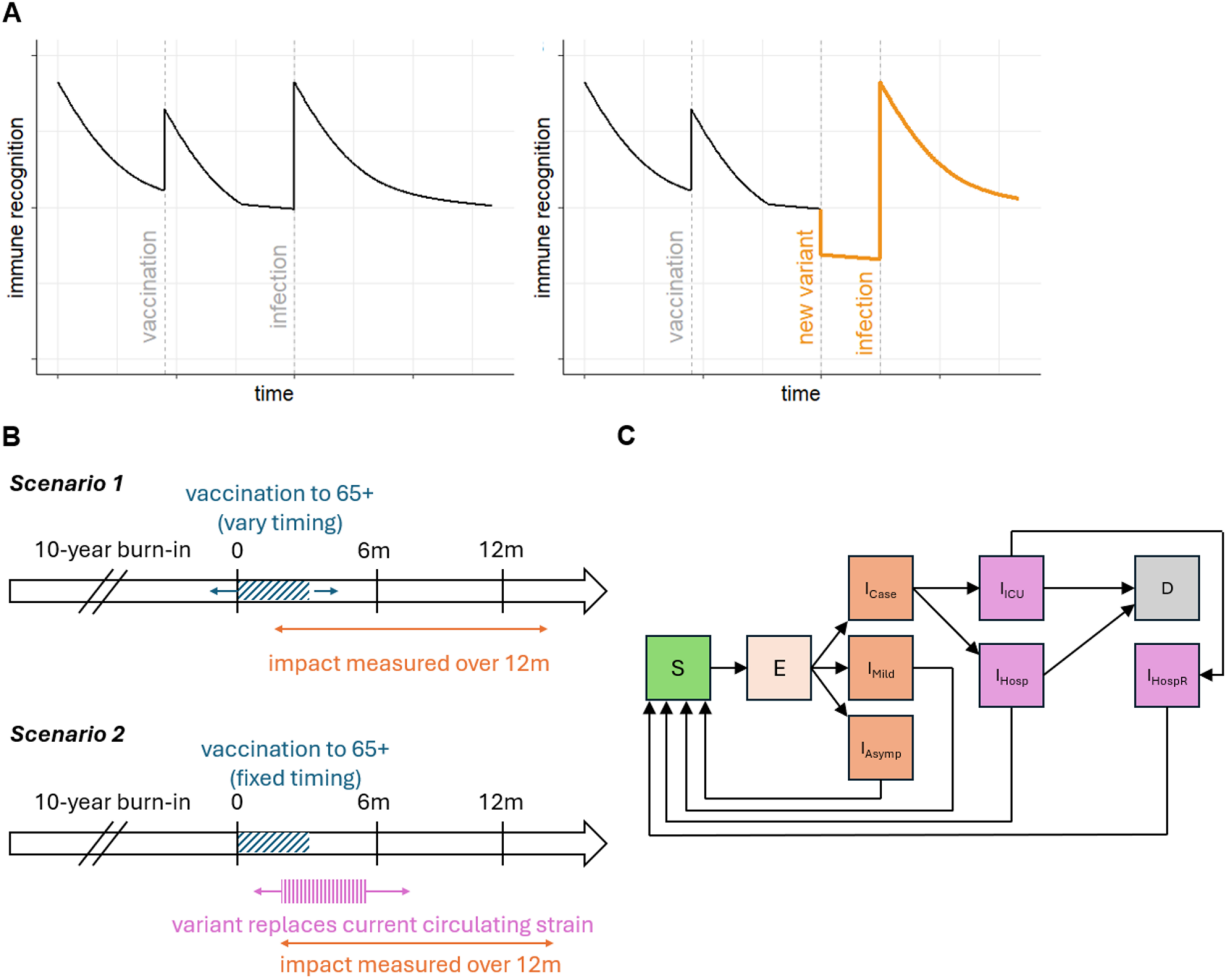
Model schematic and scenarios schematic diagram. (**A**) Schematic representation of total immune recognition over time at the individual level (formulated as the maximum of vaccine- and infection-induced recognition), following vaccination, and then infection. The right-hand side panel additionally shows the drop in immune recognition following emergence of a distinct variant, illustrated in orange. (**B**) Schematic representation of the vaccination scenarios implemented using the individual-based population-level model. (**C**) Schematic flow diagram illustrating the compartmental structure of the population-level SARS-CoV-2 model. Compartments are S (susceptible), E (exposed), I_Case_ (infectious case, prior to hospital admission), I_Mild_ (infectious mild), I_Asymp_ (asymptomatic infection), I_ICU_ (infection requiring ICU care), I_Hosp_ (infection requiring hospital admission), I_HospR_ (recovered following discharge from hospital), and D (death)

At each exposure- or vaccine-induced boost, we assume that the level of immune recognition returns to the maximum level for that type of boost (Fig. 1). Vaccine- and infection-derived immune recognition are modelled independently, but the level of protection for an individual at each timepoint is assumed to be the maximum of the two. The immune recognition model is explained in the Supplementary Information.

### Population-level transmission model and setting

We embedded this individual-level function within a stochastic model of SARS-CoV-2 transmission, severe disease, and vaccination, as previously described [19, 23]. In brief, the model stratifies the population by the following states: susceptible, exposed, infectious (either mild, asymptomatic, or requiring hospitalisation), hospitalised with or without ICU admission, and death (Fig. 1). Each modelled individual is assigned a 5-year age bin, with bin sizes corresponding to population demography. The total population size is held constant, and births and natural deaths are not simulated. In our study, we set the relative sizes of the 5-year age groups to correspond to demography for Australia [24], and parameterised age-based contacts using the mean daily number of contacts reported in POLYMOD, a large multi-country population survey, applying the United Kingdom data [25].

For each simulation, we run the model using a simulation size of 1 million agents, to balance computational efficiency with epidemiological dynamics and population heterogeneity. We initialise infection in the population with 10 infected individuals and set the remainder as Susceptible. We then run the simulation for a burn-in period of 10 years (Figure S3), to reach endemic equilibrium, before either undertaking model calibration, or simulating the specified vaccination scenarios (such that time $t=3650$ days represents the starting point for calibration or simulation). We run 20 realisations for each parameter set. We aim for the simulated levels of infection and immunity in the population after the burn-in time to represent the current age-specific SARS-CoV-2 prevalence and immune profile in the Australian population, in the absence of emergence of any new variant, and use this simulated endemic profile to compare to known epidemiological characteristics (in the calibration) and to explore future vaccination scenarios.

Model parameters are summarised in Table 1.

**Table 1:** Summary of fixed SARS-CoV-2 transmission model parameters. Additional epidemiological model parameters are outlined in Table S2

Category	Parameter	Description	Value (range tested)	Reference
Immunological model	$\mu$	Baseline level of immune recognition^1,2^	0.22	[18]
	${\delta}_{I}$	Maximum fold-change difference in immune recognition, relative to baseline, following infection	3 (1–5)	Calibrated
	${\delta}_{V}$	Maximum fold-change difference in immune recognition, relative to baseline, following vaccination	2 (1–3)	Calibrated
	${n}_{{I}_{max}}$	Maximum value of infection-induced immune recognition	${n}_{{I}_{max}}={\delta}_{I}\mu$	Calculated
	${n}_{{V}_{max}}$	Maximum value of vaccine-induced immune recognition	${n}_{{V}_{max}}={\delta}_{V}\mu$	Calculated
	${h}_{s}$	Half-life of immune recognition decay (short) (days)	35	[18]
	${h}_{l}$	Half-life of immune recognition decay (long) (days)	1000	Estimated
	${t}_{s}$	Time period for switching (days)	75	[18]
	${{n}_{50}}_{1}$	Immune recognition relative to convalescent required to provide 50% protection from mild disease	0.091	[18]
	${n}_{{50}_{2}}$	Immune recognition relative to convalescent required to provide 50% protection from hospitalisation	0.021	[18]
	$k$	Shape parameter	2.5	Estimated
	${\sigma}_{i}$	Standard deviation of individual responses	0.44	[20]
Setting	${R}_{0}$	Annual mean reproduction number	3 (3–5)	Estimated
	${\beta}_{0}$	Transmission coefficient	Calculated from ${R}_{0}$	-
	${b}_{1}$	Amplitude of seasonal forcing	0.04 (0–0.1)	Calibrated
	$c\left(a,{a}^{{\prime}}\right)$	Age-structured contact matrix	squire::get_mixing_matrix (country = “United Kingdom”)	[25]
	-	Demography	From World Population Prospects 2020 data for Australia	[24]
Vaccination	$\kappa$	Maximum coverage within age groups targeted	40% (20%, 60%)	[14, 15]
	-	Rollout period	Uniform over 3 months	-
	-	Age groups targeted (years)	65+	[12]
Simulation	N	Simulated population size	1 million	-
	${\lambda}_{\mathrm{E}\mathrm{x}\mathrm{t}\mathrm{e}\mathrm{r}\mathrm{n}\mathrm{a}\mathrm{l}}$	Number of daily importations per million population size	$1\times{10}^{-6}$	-
	-	Repetitions	20	-
	-	Burn-in period	10 years	-

^1^calculated as the estimated level of immunity against Delta for the Moderna vaccine third dose of 1.13 from Hogan et al. [18], divided by the estimated fold reduction of 5.1

^2^note that all immune recognition level values are expressed as values relative to a theoretical convalescent individual, from Khoury et al. [20]

### Model calibration approach

We previously parameterised the immunological model by fitting to vaccine product-specific effectiveness data from the United Kingdom, in the context of the Delta and Omicron variants [18]. However, more recent data highlights some limitations with this approach that we aim to address in this study.

First, the time period of observation following the third vaccine dose in the UKHSA was short, and therefore the decay of vaccine effectiveness following the first booster dose was not well captured [18]. Contemporary immunogenicity data suggests a more stable longer-term antibody response following one or more booster doses [26–28]. Here, we therefore retain the fitted immunological parameters in Hogan et al. corresponding to the initial phase of rapid decay [18], and adjust the shape parameter and parameters corresponding to the long period of slow decay to produce a longer half-life (Figure S2).

**Fig. 2:**
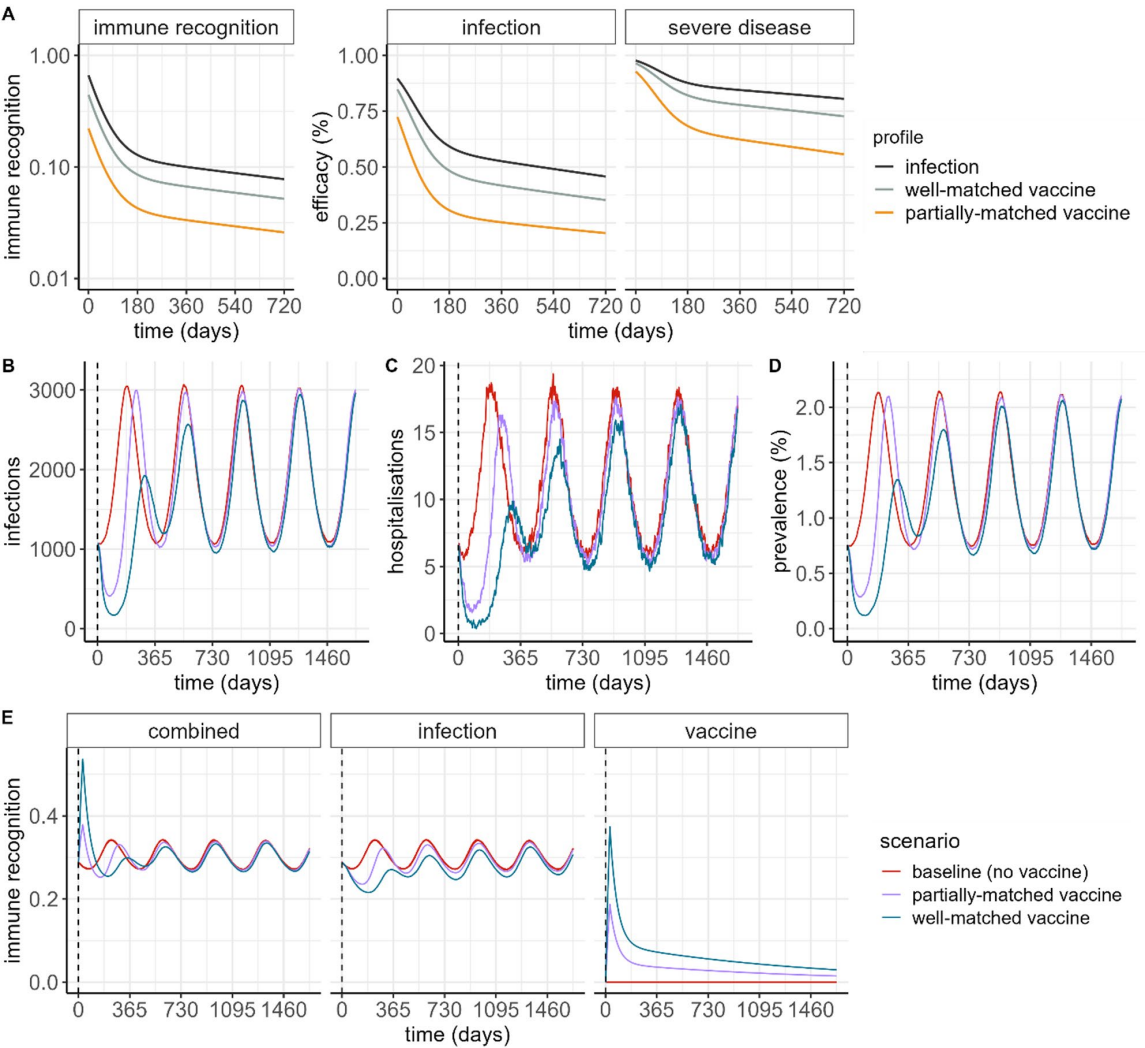
Illustration of population-level impact of individual-level immune recognition and efficacy profiles. (**A**) Individual-level immune recognition, and corresponding efficacy against infection and severe disease, following infection (black line), and following delivery of a well-matched vaccine (grey line), and a partially-matched vaccine (orange line) at day 0, relative to a naïve individual. (**B**) Daily infections, and (**C)** daily hospitalisations, per million population, following a simulation burn-in period of 10 years, and in the absence of any new variant emergence. (**D**) Prevalence of SARS-CoV-2 infection, corresponding to the epidemic trajectories in panels B and C. Prediction intervals for panels B, C, and D are visualised in Figure S6. (**E**) Population-level immune recognition. In panels B–E, the vertical dashed line in each panel represents the beginning of the simulated vaccine introduction, where a single vaccine dose is delivered to 80% of the population 15 years and older, over a 2-month period. Note that high vaccination coverage (80%) is used in these illustrative simulations to demonstrate the impact of the immune recognition and efficacy mechanism applied in the individual-level transmission model

Second, the initial immunity model was calibrated to a particular category of exposure (i.e. vaccination with a vaccine antigen mismatched to the virus antigen). We assume that the fitted parameters represent efficacy for an ancestral strain-matched vaccine against the Omicron variant (i.e. a significant mismatch) [18]. We introduce a new scaling parameter $\delta$, which corresponds to the maximum fold-change difference in immune recognition relative to the baseline fitted level of immune recognition, immediately following either infection or vaccination (see Fig. 2). We define ${\delta}_{V}$ as the fold-change increase for a vaccine (where the value can be different depending on the level of strain-matching), and ${\delta}_{I}$ as the fold-change increase for infection, and test values for $\delta$ between 1 and 5, where ${\delta}_{I}>{\delta}_{V}$.

Third, the immune profile of the population not receiving a recent booster dose is now markedly different to early 2022, due to repeated infections. To account for this change in epidemiology, we estimate the difference between infection- and vaccine-induced immune protection using vaccine effectiveness data.

### Model calibration procedure

For the population-level transmission model, we did not explicitly fit the model to time series of reported cases or severe outcomes. With COVID-19 testing and surveillance now limited, notified cases are not representative of underlying community transmission. Additionally, epidemic wave timing has been determined both by waning immunity, and the emergence of new distinct variants [29], which means that the available time series are not necessarily reflective of the endemic transmission dynamics the underlying model is aiming to capture. We instead aimed to calibrate the model to contemporary broad epidemiological characteristics.

In the model calibration, we aim to capture the following representative characteristics: (a) an annual population-level attack rate between 0.5 and 1 (representing that on average, individuals are infected every 1 to 2 years); (b) an annual seasonal peak in modelled hospitalisations of a magnitude similar to the reported annual peak hospitalisations at the state level in Australia; and (c) endemic transmission throughout the year, with a seasonal peak between two and three times higher compared to the daily infections between epidemics. These characteristics are fully described in the Supplementary Information. To capture these features, we first simulate the population-level transmission model in the absence of vaccination, simultaneously varying the mean reproduction number ${R}_{0}$, the amplitude of seasonal forcing ${b}_{1}$ and the fold-change increase in infection-induced immune recognition ${\delta}_{I}$. By visual inspection, we select the parameter set that most closely aligns with the specified characteristics. The parameter ranges are outlined in Table 1 and S2. As an additional verification, following calibration of the transmission model, we compare the modelled infection hospitalisation ratio (IHR) by age with IHR estimates from the 2024 UK winter COVID-19 Infection Survey [30].

Second, we implement vaccination in the model to a fixed cohort of individuals, varying the relative fold-change increase in vaccine-induced immune recognition ${\delta}_{V}$. We use the model outputs to estimate the observed population-level vaccine effectiveness that is produced by the model over a six-month window, and select a ${\delta}_{V}$ that produces an observed population-level impact against infection of approximately 33% over 6 months for a well-matched vaccine (guided by the recent vaccine effectiveness estimate for the Monovalent XBB.1.5 booster of 32.7% (16.1–46.0%) in the UK SIREN healthcare worker cohort [31]).

### Setting and scenarios explored

We first explore the impact of routine COVID-19 vaccination, assuming no new antigenically distinct variant emergence. To do this, we simulate the rollout of a COVID-19 vaccine booster dose distributed at a constant rate over a 3-month period, varying the level of coverage, and estimated the impact in terms of averted infections and hospitalisations over 12 months. We assume a central coverage value of 40% (based on recent Australian COVID-19 booster dose coverage data estimates of 34% (65–74-year-olds) and 50% (75+ years) [15]) and simulate additional values of 20% and 60%. We explore two vaccine delivery programs: either a single dose delivered to the 65+ years population, or a single 65+ years dose with an additional booster dose to the 75+ years population after 6 months. We also explore the impact of varying vaccination timing (either before or after the start of an annual wave of infection), and of the degree of vaccine matching to the circulating variant.

Second, we explore the impact of routine COVID-19 vaccination, in the context of emergence of a new distinct variant of concern. We set the default value of VFR to 1.5, such that the magnitude of immune escape would be similar to that from Delta to Omicron, and explore an additional value of 2, with the variant replacing the current circulating strain over a time period of 60 days. We then explore allowing the timing of variant emergence to vary relative to vaccination. These scenarios are summarised in Fig. 1B.

#### Data availability

The R package used to undertake the COVID-19 model simulations is available with a description at https://github.com/abhogan/safir_immunity. All code to replicate the analysis and figures in this paper is available at https://github.com/abhogan/covid_endemic_vaccine.

Ethical approval was obtained from the UNSW Sydney HREAP Executive (iRECS6005).

## Results

### Model calibration

We found that for an annual mean reproduction number of ${R}_{0}=3$ and a relative fold-change in immune recognition from infection of ${\delta}_{I}=3$, combined with an amplitude of seasonal forcing of ${b}_{1}=0.04$, the modelled annual attack rate was approximately 0.7 (corresponding to approximately two-thirds of the population being infected each year), with an annual hospitalisation epidemic peak corresponding to around 20 per million per day, in line with our calibration criteria (Figure S4). Increasing both the transmission level and immunity following infection simultaneously (i.e. ${R}_{0}$ and ${\delta}_{I}$) produced similar attack rates, therefore we retained the parameter combination of ${R}_{0}=3$ and ${\delta}_{I}=3$ for the main simulations. The model produced annual epidemic waves of infection, and an average annual prevalence of approximately 1.4% (Fig. 2B–D). We found that the modelled IHR aligned reasonably well with estimates of IHR from the UK COVID-19 Infection Study (Figure S5) [30].

We estimated that ${\delta}_{V}=1$corresponded to vaccine effectiveness against infection over six months of approximately 21% (denoted “unmatched vaccine”), and ${\delta}_{V}=2$ corresponded to vaccine effectiveness over six months of approximately 36% (denoted “well-matched vaccine”). We categorised ${\delta}_{V}=1.5$as corresponding to a “partially-matched vaccine”. These immune recognition and efficacy profiles are plotted in Fig. 2A. Illustrative population-level vaccine impact for the well-matched and partially-matched vaccine profiles, with vaccination delivered to ages 20+ years at 80% coverage are shown in Fig. 2B–D, with the corresponding population-level immune recognition over time plotted in Fig. 2E. Fixed and calibrated parameters are summarised in Table 1 and S2.

### Impact of routine vaccination

**Fig. 3:**
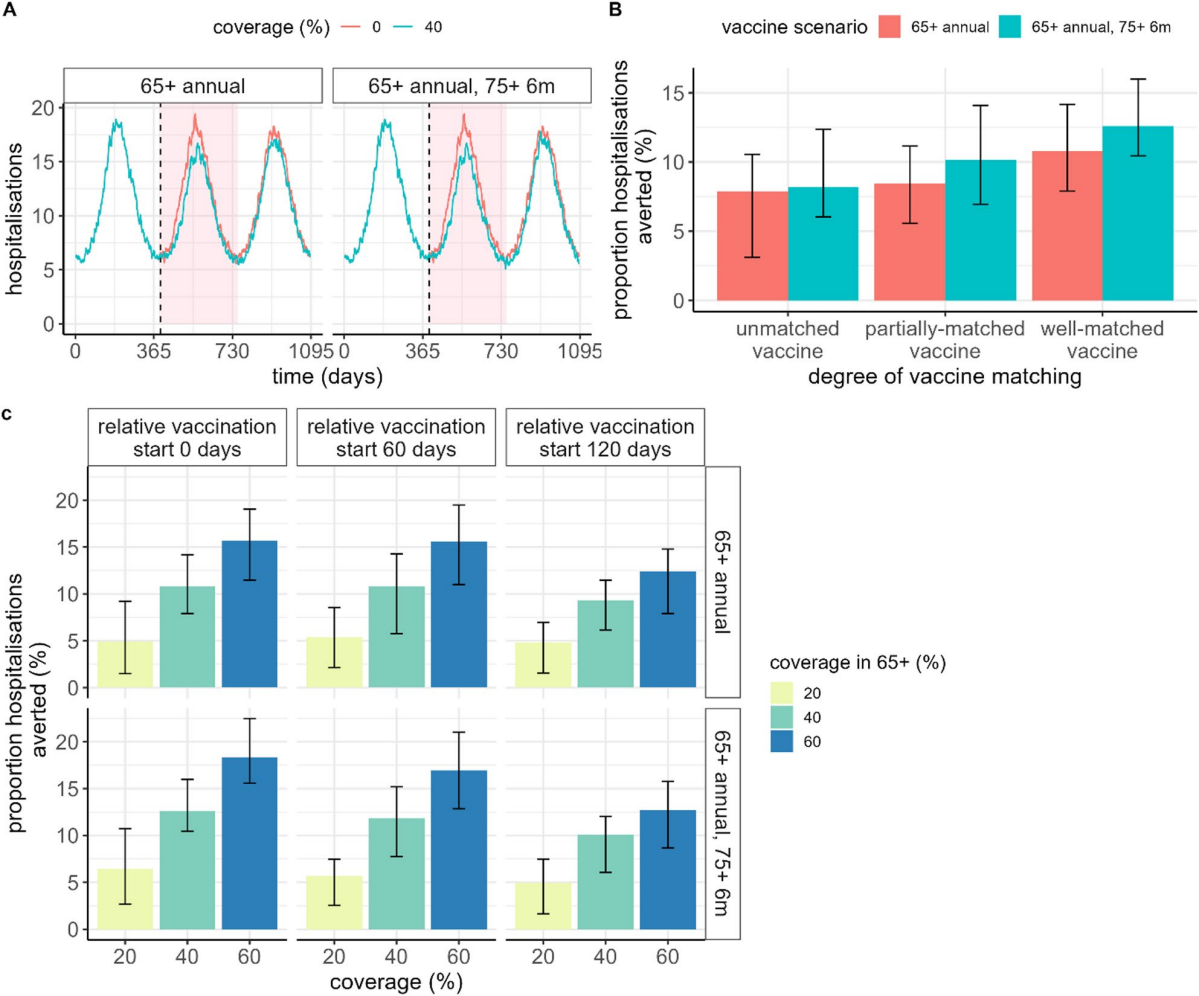
Impact of routine vaccination over a 12-month period, with no change in circulating strain. (**A**) Daily hospitalisations per million population for a scenario with no vaccination (red line), compared to a vaccine delivered at 40% coverage (blue line), where the start of vaccination delivery is shown with a vertical dashed line, and the pink shaded region denotes the time window over which events are aggregated to quantify impact. (**B**) Proportion of hospitalisations averted for three vaccine product scenarios: either an unmatched vaccine, with ${\delta}_{V}=1$, a partially-matched vaccine, with ${\delta}_{V}=1.5$, or a well-matched vaccine, with ${\delta}_{V}=2$, assuming 40% coverage. (**C**) Proportion of hospitalisations averted for coverage levels of 20%, 40% and 60%, assuming delivery of a well-matched vaccine, for different timings of vaccination commencement relative to the trough in seasonal infections, where the “0 days” scenario is reflected in panel A. In all panels, “65+ annual” refers to a single vaccine dose delivered to the 65+ years population, and “65+ annual, 75+ 6m” represents the same scenario but with an additional dose delivered to the 75+ years population after a six-month period. The black bars represent 95% prediction intervals. Additional trajectories as in panel A, but with vaccination commencing at different timepoints relative to the epidemic wave and prediction intervals visualised, are shown in Figure S7. Values are shown in Table S3

We estimated that overall, a single vaccine dose delivered to the 65+ population at 40% coverage could avert around 6%–11% of COVID-19 hospitalisations over a single seasonal epidemic wave (corresponding to 24–45 averted per 100,000 individuals), depending on the degree that the vaccine is matched to the dominant circulating strain (Fig. 3, Figure S7). Up to 8%–16% of hospitalisations may be averted if a higher level of vaccine coverage of 60% is achieved (around 36–65 averted per 100,000 individuals) (Table S3). An additional vaccine dose delivered to the 75+ population was found to be beneficial (averting up to an estimated 10 additional hospitalisations per 100,000 individuals at 60% coverage). However, for the scenarios modelled, a two-dose strategy with a partially-matched vaccine generally averted slightly fewer hospitalisations compared to a well-matched vaccine delivered to only the 65+ population (Table S3).

We demonstrated that timing of vaccine implementation relative to a seasonal wave is important. Assuming vaccination is rolled out uniformly over a 3-month period, the maximum benefit was achieved if vaccination is commenced around 6 months prior to the estimated epidemic peak, with only around two-thirds of the benefit achieved if vaccination commences 4 months later, or well into the epidemic wave (Fig. 3C, Table S3).

At the individual level, we estimated that receiving a partially matched vaccine earlier in the season was relatively comparable to a later well-matched vaccine delivered around the peak of the season, in terms of risk of hospitalisation within that epidemic season (Figure S8). However, a well-matched vaccine provided more long-lasting benefit into the subsequent COVID-19 epidemic wave (around 25% of hospitalisations avoided in the second season in those vaccinated).

### Impact of vaccination in context of new variant emergence

**Fig. 4:**
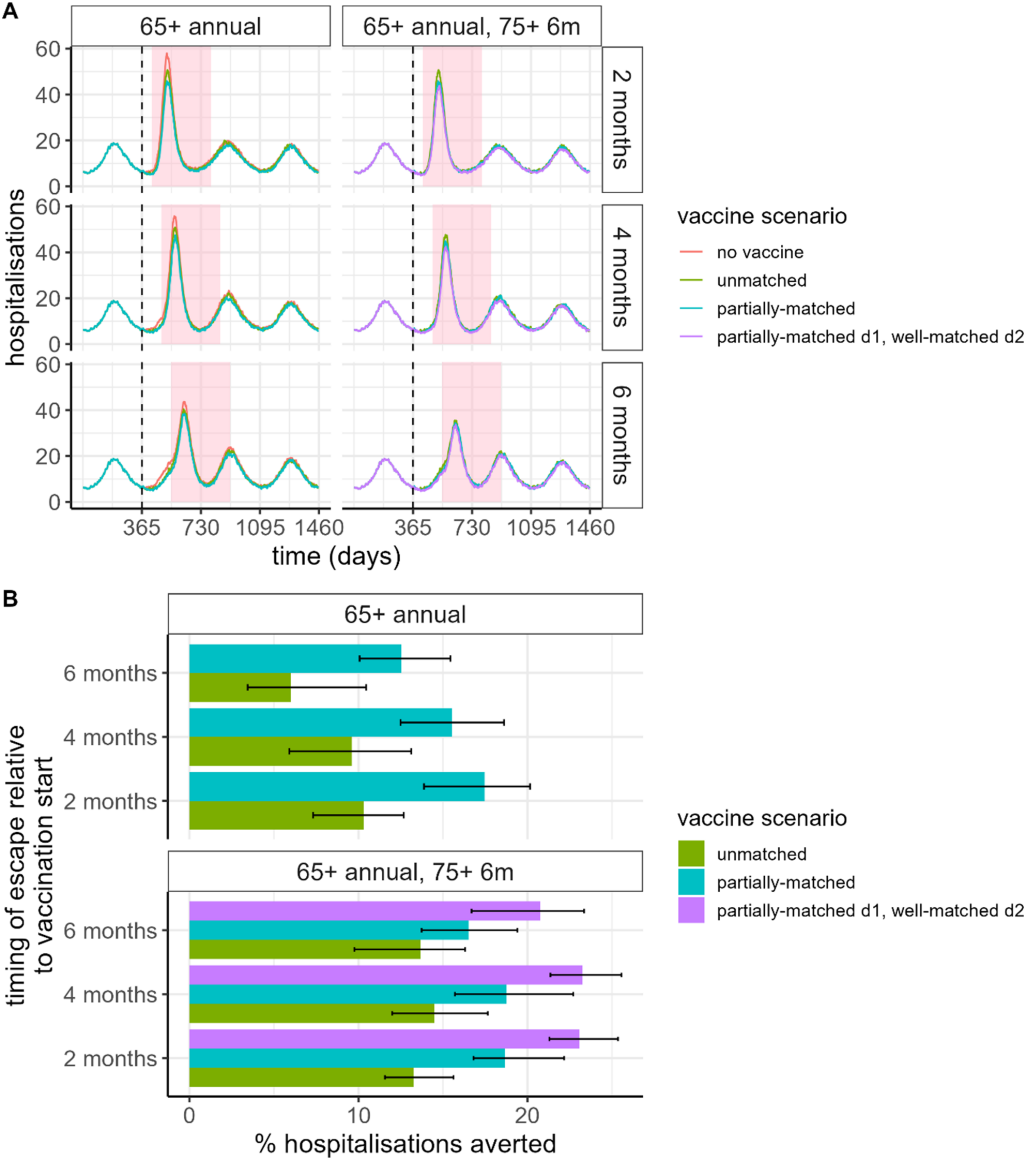
Impact of vaccination in the context of emergence of a new distinct variant strain. (**A**) Daily hospitalisations, following variant emergence at either 2, 4, or 6 months following vaccination (where the start of vaccine delivery is shown with a vertical dashed line). (**B**) Vaccine impact in terms of percentage of hospitalisations averted, where the total impact for each timing scenario is aggregated over a 12-month period following variant emergence (shown as the pink shaded regions in panel A). Coloured lines and filled bars represent four scenarios: no vaccine (red); an unmatched vaccine (green), with $\delta=1.5$, a partially-matched vaccine, with $\delta=2$ (teal), and a partially matched first dose to the 65+ years population, with a second dose after 6 months delivered to the 75+ years population with a vaccine dose that is well-matched to the new variant strain (purple). The black bars represent 95% prediction intervals. Coverage is assumed to be 60%. Values are shown in Table S4

We found that even a poorly matched vaccine delivered to 60% of the eligible population provides considerable population-level benefit if a new variant emerges (Fig. 4, Figure S9, and Figure S10). If a second vaccine dose is delivered to the 75+ population 6 months after the first dose, at the same coverage, close to 20% of hospitalisations could be averted. Although the total population-level impact was estimated to be higher if the second dose is updated to match the new variant strain, an additional dose to the highest risk group with the same vaccine could potentially avert up to 30% more hospitalisations than a single-dose policy (Table S4).

## Discussion

To date, mathematical models of SARS-CoV-2 transmission and vaccination have largely been formulated in the context of pandemic COVID-19, whereas the present need is to inform longer-term COVID-19 vaccination policies. In our study, we refined a model of individual-level immunity to incorporate the most recent evidence on the effectiveness of vaccines and hybrid immunity. Using this model, we captured contemporary endemic patterns of SARS-CoV-2 infections and hospitalisations observed in Australia and quantified the benefits from variations to an ongoing COVID-19 booster program in older adults. We demonstrated that vaccine coverage and the degree that a vaccine is matched to the current circulating strain are key determinants of COVID-19 vaccine benefit. Of the strategies explored, our results suggest that increasing uptake of the recommended annual booster dose in older adults will have the largest impact in reducing COVID-19 hospitalisations. While optimising the timing of a booster dose relative to the seasonal wave can modify impact, receipt of a vaccine at any time during the season is beneficial.

In Australia, booster doses are now recommended every 12 months for the 65+ years population, and every 6 months for the 75+ years population [12]. Uptake has generally declined over time; around 43% of individuals aged 75+ years received a COVID-19 booster dose over the 12 months to January 2025, and in individuals 18+ years, around half as many booster doses were delivered in 2024 compared to 2023 [32–34]. We found that a partially matched vaccine delivered once per year to the 65+ years age group at 40% coverage likely averts about 10% of COVID-19 hospitalisations across the population, relative to a no-vaccination scenario. An additional booster to the 75+ population after 6 months can deliver targeted protection to the age group at highest risk of severe disease, and with higher coverage, a well-matched vaccine can avert around 20% of hospitalisations relative to a scenario without vaccination.

We did not explicitly include historical vaccine uptake in the baseline calibrated model, because we assumed that the impact of prior vaccination is reflected in the observed epidemiological data. While this assumption may have affected our baseline parameter estimates in overall transmission and the magnitude of seasonality, the effect is likely to be small. Further, accounting for historical vaccine uptake and impact in the model would have added considerable complexity and was unlikely to have materially changed our estimates of future vaccine impact, noting that the objective of our study was to understand the relative benefit of different potential strategies. Because our model captured the impact of infection and vaccination upon an already highly exposed population who had received at least the primary series of a COVID-19 vaccine, we did not explicitly capture how values of the boost to immune recognition may vary depending on exposure or immunisation history.

In our study we examined the importance of both timing and coverage, and found that for realistic values of these parameters, achieving higher coverage with a booster dose program was more influential in averting hospitalisations compared to the timing of vaccine delivery in advance of the COVID-19 season. In Australia, doses are typically delivered throughout the year, with eligibility based on time since last booster, and although uptake tends to increase with SARS-CoV-2 activity [32], attempting to control timing through policy may be difficult. Instead, our study indicates that strategies that focus on increasing booster dose uptake in older Australians may be both more effective and efficient in reducing the COVID-19 disease burden. With novel combination vaccines now in the development pipeline [35, 36], enabling concurrent protection against multiple respiratory viruses, there may be future opportunities to efficiently increase COVID-19 vaccine booster dose coverage.

While SARS-CoV-2 evolution is unpredictable, even the emergence of novel antigenic variants such as JN.1 have not led to disease burdens comparable to waves between 2020 and 2022, likely because of high existing individual and population-level immunity, particularly to disease consequences of infection [37]. However, the emergence of an antigenically distinct variant still has the potential to cause an unanticipated, rapid epidemic wave. In our study, we found that in the event of such an occurrence, vaccine impact is substantially increased, even if a new variant emerges 6 months after vaccine delivery, noting that the benefit of COVID-19 booster doses would vary depending on the characteristics of a novel variant. While we focussed on individual-level immune recognition in this analysis, this framework can be used to examine other consequences of SARS-CoV-2 evolution, including transmissibility and virulence.

Our study has several limitations. First, in terms of calibration, we elected to take a qualitative approach in requiring the model to mimic observed trends in seasonal peaks in hospitalised cases, and the estimated annual incidence rate. This was chosen instead of calibration to case time series data because of uncertain temporal changes in case definitions and testing practices over the period of interest, along with impacts of novel variants, which the model excludes. If available, representative population-level surveys of respiratory pathogen prevalence would help inform model calibration. Second, while the longer-term rate of decay in the previously fitted immunity model was not well-characterised, the calibration source has not been updated with sufficient regularity to facilitate refitting. We therefore revised our immunity model calibration approach and used multiple data sources to validate the underlying immunity profile for protection following exposure, in order to represent a more durable long-term immune response. Other profiles of longer-term immunity may also be plausible. Third, by design our calibrated model produces a single annual (winter) peak, whereas in some settings, including in Australia, an additional smaller (summer) wave has previously been observed. However, given this is based on only two years of observations, and out-of-season waves have been at least partially driven by the emergence of new distinct variants, stability of this pattern is not yet established.

Now that we have moved beyond pandemic-era COVID-19, it is timely for the benefit of vaccine programs to be assessed, as they are integrated into routine national immunisation programs. This model of SARS-CoV-2 transmission and vaccination more accurately captures recent data on protection derived from infection and vaccination and reproduces temporal patterns of the magnitude currently observed in Australia. As demonstrated here, the model can be used to quantify the benefits of ongoing COVID-19 vaccination programs in priority and higher risk groups. This model and calibration approach could be readily applied to other settings (either locally or internationally), by varying the transmission, seasonality, and demography parameters. Specifically, this approach could be used to explore vaccination strategies and provide valuable policy insights for specific Australian jurisdictions or more refined settings (for example, metropolitan versus regional population), by drawing on surveillance data and expert knowledge in collaboration with health policy partners. There is a need for future work to consider alternative temporal dynamics – it is possible that other epidemic patterns may emerge, and may differ between climatic regions. Finally, with combined vaccines that simultaneously target multiple respiratory pathogens now in development [35, 36], dynamic models that capture individual and population immunity across multiple viruses will be required to help determine the most efficient and effective vaccine programs to reduce respiratory disease burden. 

## Supplementary Information

Below is the link to the electronic supplementary material.

Supplementary Material 1

## Data Availability

The R package used to undertake the COVID-19 model simulations is available with a description at https://github.com/abhogan/safir_immunity. All code to replicate the analysis and figures in this paper is available at https://github.com/abhogan/covid_endemic_vaccine.

## Abbreviations

COVID-19: Coronavirus disease
SARS-CoV-2: Severe acute respiratory syndrome coronavirus 2
WHO: World Health Organization
UK: United Kingdom
UKHSA: United Kingdom Health Security Agency
WHO SAGE: World Health Organization Strategic Advisory Group on Immunization
VFR: Variant fold reduction
AR: Attack rate
