# Supplementary material for: Capturing SARS-CoV-2 immune landscapes to inform future strategies for COVID-19 vaccination in a high-income setting: a mathematical modelling study
Source: BMC Infect Dis. 2026 Mar 25;26:884. doi: 10.1186/s12879-026-13074-3 (PMC13137643; doi:10.1186/s12879-026-13074-3)
Supplement: Supplementary file 1 — Supplementary Material 1 [file 12879_2026_13074_MOESM1_ESM.pdf]

Supplementary material

Capturing SARS-CoV-2 immune landscapes to inform future strategies for COVID-19 vaccination in a high-income setting: a mathematical modelling study

Alexandra B Hogan[1], David J Muscatello[1], Bette Liu[1,2], Gemma Nedjati-Gilani[3], James G Wood[1]

1 School of Population Health, Faculty of Medicine and Health, UNSW Sydney, Australia

2 National Centre for Immunisation Research and Surveillance, Sydney, Australia

3 MRC Centre for Global Infectious Disease Analysis, Imperial College London, London, UK

S1. Additional methods

Immunological model

An individual's level of immunity is comprised of infection-induced and vaccine-induced immune recognition. Each of these types of immunity are tracked independently at the individual level, and can be boosted to different thresholds, but follow the same dynamics of decay over time. This model is derived from Khoury et al (1), and is described in the context of the population-level model in Hogan et al (2,3).

The maximum values of infection-induced and vaccine-induced immune recognition (denoted by I and V subscripts respectively), achieved immediately after infection or vaccination, are estimated as part of the model calibration process, and the mean values of these types of immune recognition are defined as

$$n_{I_{\max}} = \delta_I \mu \qquad (1)$$

and

$$n_{V_{\max}} = \delta_V \mu \qquad (2)$$

where the parameter μ represents the immune level estimated in (2), here referred to as the "baseline" level of immune recognition (see Table 1), and δ_I and δ_V correspond to the maximum fold-change difference in immune recognition, compared to the baseline estimated level of immune recognition, achieved immediately after infection and vaccination respectively. The values of δ_I and δ_V are estimated during model calibration.

Each category of immune recognition decays over time according to the biphasic exponential functions

$$n_I(t) = n_{I_{\max}} \Lambda(t) \qquad (3)$$

and

$$n_V(t) = n_{V_{\max}} \Lambda(\mathrm{t}), \qquad (4)$$

where

$$\Lambda(t) = \frac{\exp(\pi_1 t + \pi_2 t_s) + \exp(\pi_2 t + \pi_1 t_s)}{\exp(\pi_1 t_s) + \exp(\pi_2 t_s)}, \quad (5)$$

and where n_x is the maximum level of immune recognition for either infection or a vaccine, $\pi_1 = -\ln(2)/h_s$ is the rate for the initial period of fast antibody decay; $\pi_2 = -\ln(2)/h_l$ is the rate for the period of slow decay; t is the time since the last infection or vaccine dose; and t_s is the period of switching between the fast and slow decays.

Upon either recovery from infection, or following vaccination, an individual's immune recognition returns to the maximum level (i.e. the value of $\Lambda(t)$ is reset to 1 for either infection or vaccination), and the decay process recommences. Individual-level variation is accounted for in that each time either an infection or vaccine event occurs, the value of $n_{I_{\max}}$ or $n_{V_{\max}}$ is drawn from a Normal distribution with standard deviation σ_i. Note that we assume no difference in immune recognition by age, nor any persistent difference between individuals (i.e. n_I and n_V are resampled at each infection and vaccine event).

Upon emergence of a new distinct variant strain, immune recognition is reduced linearly over a two-month window, such that after this time window, immune recognition is reduced by a scaling factor termed the "variant fold reduction", or VFR (initially described in Hogan et al (3)), where

$$n_I(t) = \frac{n_I}{\text{VFR}} \Lambda(t) \quad (6)$$

and

$$n_V(t) = \frac{n_V}{\text{VFR}} \Lambda(t). \quad (7)$$

For a vaccine dose that is matched to the new variant strain, the VFR is divided by a scaling factor ψ to account for the additional effectiveness of an adapted vaccine. Following infection with a new variant strain, the level of infection-induced immune recognition returns to the maximum level without VFR scaling (i.e. to represent well-matched infection-induced immunity against the current circulating strain).

At each timestep, the total level of immune recognition n_T is calculated as the maximum of the infection- and vaccine-induced levels, such that

$$n_T = \max(n_I, n_V). \quad (8)$$

We then assume a logistic relationship between the total level of immune recognition n_T and effectiveness ϵ_m, such that

$$\epsilon_m(n_T(t)) = \frac{1}{1 + e^{-k[\log_{10}(n_T(t)) - \log_{10}(n_{50_m})]}}, \quad (9)$$

where k is the shape parameter and n_{50_m} is the immune recognition required to provide 50% protection against each endpoint (infection ($m = 1$) and hospitalisation ($m = 2$)).

In the model, we apply the effectiveness against hospitalisation conditional on being infected. This is calculated as

$$\epsilon_2(n_T(t))_{\text{COND}} = 1 - \frac{1 - \epsilon_2(n_T(t))}{1 - \epsilon_1(n_T(t))}. \quad (10)$$

The parameters and their values are described in Table 1.

Transmission model

We adapted an existing individual-based population model of SARS-CoV-2 transmission and vaccination ("safir"), to capture the modified immunological model (3). The compartmental model structure is identical to that previously described, but we provide a summary of the model below.

The model stratifies the population into the following epidemiological states (3):

- S = uninfected
- E = exposed to infection but not yet infectious
- I_{Mild} = infected and infectious with mild symptomatic infection that does not require hospitalisation
- I_{Asymp} = infected and infectious with asymptomatic infection
- I_{Case} = infected and infectious with disease that will require hospitalisation
- I_{Hosp} = cases that have been hospitalised in a general ward bed
- I_{ICU} = cases that have been admitted to an intensive care unit (ICU)
- I_{HospR} = cases that have been stepped down from ICU into a general ward bed for recovery
- D = cases that have died.

In the transmission model, infection is initiated into the population at the start of the simulation (with a seed of 10 infections). The model is run with a discrete time step of 0.25 days across a simulation size of 1 million agents. We additionally include a constant external force of infection, representing external infections from outside the modelled (closed) population. All state durations are modelled using an Erlang distribution with shape parameter 2. Mean state durations and branching fractions are summarised in Table S1, with parameter definitions in Table S2.

Table S1: Transmission model state transitions. ICU: intensive care unit. All state durations are modelled using an Erlang distribution with shape parameter 2. Table is derived from that presented in Hogan et al (3). Parameters and functions are defined in Table S2.

**Event**	**State transition**	**Branching fraction or probability**	**Mean state duration**
Infection	$S \rightarrow E$	Driven by immunity and force of infection	N/A
Latent to asymptomatic infection	$E \rightarrow I_{\text{Asymp}}$	$\phi_0(1-\phi_1(a))$	$1/\alpha$
Latent to mild symptomatic infection	$E \rightarrow I_{\text{Mild}}$	$(1-\phi_0)(1-\phi_1(a))$	$1/\alpha$
Latent to severe infection	$E \rightarrow I_{\text{Case}}$	$\phi_1(a)(1-\epsilon_2(n_{T(t)})_{\text{COND}})$	$1/\alpha$
Severe case to ICU	$I_{\text{Case}} \rightarrow I_{\text{ICU}}$	$\phi_2(a)$	$1/\gamma_2$
Severe case to hospital	$I_{\text{Case}} \rightarrow I_{\text{Hosp}}$	$(1-\phi_2(a))$	$1/\gamma_2$
ICU to death	$I_{\text{ICU}} \rightarrow D$	$\mu_2(\text{a})$	$1/\gamma_{4,0}$
Hospitalised to death	$I_{\text{hosp}} \rightarrow D$	$\mu_1(\text{a})$	$1/\gamma_3$

ICU to recovered general ward bed	$I_{ICU} \rightarrow I_{HospR}$	$(1 - \mu_2(a))$	$1/\gamma_{4,1}$
ICU recovered to susceptible	$I_{HospR} \rightarrow S$	NA	$1/\gamma_5$
Hospitalised to susceptible	$I_{Hosp} \rightarrow S$	$(1 - \mu_1(a))$	$1/\gamma_3$
Symptomatic case to susceptible	$I_{Mild} \rightarrow S$	NA	$1/\gamma_1$
Asymptomatic case to susceptible	$I_{Asymp} \rightarrow S$	NA	$1/\gamma_1$

We assume that only infections in the community contribute to onward infection (i.e. the states I_{Asymp}, I_{Case}, and I_{Mild}) and that social mixing is governed by an age-structured contact matrix $c(a, a')$. The total force of infection acting on each individual is given by

$$\lambda_{\text{Total}} = \lambda_{\text{External}} + \lambda(a, n_T, t) \tag{11}$$

where

$$\lambda(a, n_T, t) = \beta_0 \left[1 + b_1 \left(\cos\left(\frac{2\pi t}{365}\right)\right)\right] [1 - \epsilon_1(n_T(t))] \sum_{a'} c(a, a')[I_{\text{Asymp}}(a', t) + I_{\text{Mild}}(a', t) + I_{\text{Case}}(a', t)] \tag{12}$$

and β_0 represents the transmission coefficient, b_1 represents the amplitude of seasonal forcing, ϵ_1 represents efficacy against infection, and $n_T(t)$ represents the total level of immune recognition.

Model calibration

As outlined in the main text, we aimed to calibrate the model so as to reproduce characteristics relating to (a) population-level attack rate; (b) the magnitude of epidemic hospitalisations peaks; and (c) endemic infections, using available Australian surveillance data. These three calibration characteristics are described in more detail as follows.

Annual population-level attack rate. Surveillance data that can be used to calculate the incidence or prevalence of SARS-CoV-2 infection are not routinely collected in any jurisdiction. While serosurveys were conducted across 2022 (4), these were not continued into the more relevant post-Omicron era of 2023 and 2024. As such, in order to calibrate the level of transmission in the model, we relied on data from the UK Covid-19 Infection Survey and estimated that about 25% of the population were infected over the 2023–24 winter wave (a wave that was comparable in size to two previous waves in the same 12 month period) suggesting that based on longer-term sustained levels of SARS-CoV-2 infection in the UK, an annual attack rate of between 0.5–1 (i.e. individuals infected between every one and two years) is appropriate (5).

Seasonal hospitalisations. We obtained, where possible, time series hospitalisations data from Australia by state over the years 2023–24. Data from Queensland Health on the number of persons hospitalised with COVID-19 indicates a 2024 winter peak in hospitalisation occupancy of approximately 350-400 persons (6). Assuming a Queensland population of approximately 5.2 million and an average hospital stay of 5 days, this translates to a seasonal peak of around 15 hospital admissions per million per day. Using similar data from Government of Western Australia Department of Health (where data points to a maximum hospital occupancy of approximately 200–250 persons) (7), a state with a population of approximately 2.5 million

people, the data translates to a seasonal peak of around 20 hospital admissions per million per day. Finally, data from Victoria, a state with a population of 7 million, indicates a peak seasonal COVID-19 hospital occupancy of about 350–450 (8), translating to around 15 hospital admissions per million per day. We therefore aimed to reproduce maximum daily hospitalisation incidence of 15 to 20 per million population.

Infection characteristics. SARS-CoV-2 infection remains very common, and while seasonal epidemics occur, transmission of the virus is now generally sustained throughout the year (see, for example, UK respiratory virus surveillance data, indicating a baseline level of SARS-CoV-2 positive tests being maintained throughout the year (9), and positive laboratory confirmation data from the Australian National Notifiable Diseases Surveillance System, shown in Figure S1 (10)).

Infection hospitalisation ratio. As an additional post-calibration verification of our model, we compared the age-specific infection hospitalisation data that was input into the model, alongside the modelled age-specific infection hospitalisation ratio (IHR) (representing the IHR in an exposed and previously vaccination population), with IHR estimates from the 2024 UK winter COVID-19 Infection Survey (11).

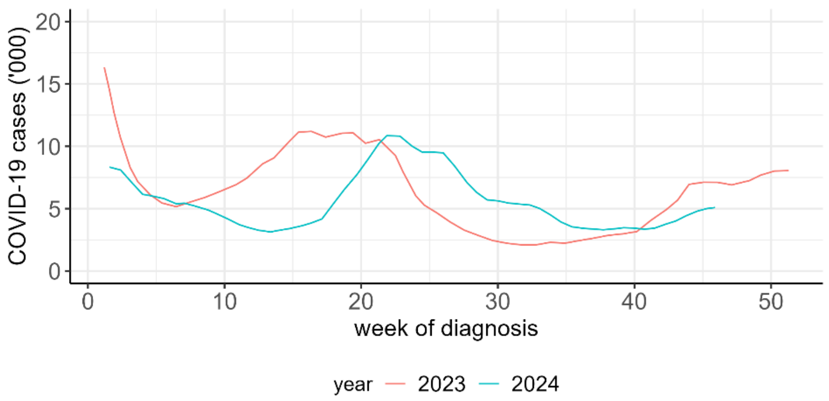

Figure S1: Number of laboratory-confirmed COVID-19 cases notified to the Australian National Notifiable Diseases Surveillance System, by year and week of diagnosis, from 1 January 2023 to 17 November 2024. Data extracted from the Australian Respiratory Surveillance Report 16 – 21 October 2024 to 17 November 2024 (10), using WebPlotDigitizer software (12).

Vaccine allocation

Age-based vaccine allocation is implemented in the transmission model, where the user specifies the target population (here the 65–69, 70–74, 75–79 and 80+ years groups), the day that vaccination commences, the rollout period for a single dose (here 90 days), and the time window between vaccine doses (here 180 days).We then use this information to calculate the number of vaccine doses to be distributed each day over the rollout window, so that doses are administered at a constant rate. Age groups are vaccinated sequentially starting with the oldest age group.

Table S2: Additional epidemiological model parameters

Parameter	Description	Value (range tested)	Reference
$1/\alpha$	Mean latent period	4.6 days	(13,14)
$1/\gamma_1$	Mean duration of mild or asymptomatic infection	7 days	(14)
$1/\gamma_2$	Mean duration of severe infection prior to hospitalisation	7 days	(14)
$1/\gamma_3$	Mean duration of hospitalisation for non-critical cases if survive or if die	9 days	Assumption
$1/\gamma_{4,1}$	Mean duration in ICU if survive	14.8 days	(13)
$1/\gamma_{4,0}$	Mean duration in ICU if die	11.1 days	(13)
$1/\gamma_5$	Mean duration in recovery after ICU	3 days	Assumption
$\mu_1(a)$	IFR in the absence of immunity (i.e. for a naïve individual) for non-ICU	Age-dependent	Table S2 in Hogan et al 2021 (13)
$\mu_2(a)$	IFR in the absence of immunity (i.e. for a naïve individual) for ICU	Age-dependent	Table S2 in Hogan et al 2021 (13)
ϕ_0	Proportion of mild infections that are asymptomatic	0.2	Estimated
$\phi_1(a)$	Probability of developing severe disease requiring hospitalisation in the absence of immunity	Age-dependent	Table S2 in Hogan et al 2021 (13)
$\phi_2(a)$	Proportion of hospitalisations requiring ICU	Age-dependent	Table S2 in Hogan et al 2021 (13)

References

1. Khoury DS, Cromer D, Reynaldi A, Schlub TE, Wheatley AK, Juno JA, et al. Neutralizing antibody levels are highly predictive of immune protection from symptomatic SARS-CoV-2 infection. Nat Med. 2021 July;27(7):1205–11.

2. Hogan AB, Doohan P, Wu SL, Mesa DO, Toor J, Watson OJ, et al. Estimating long-term vaccine effectiveness against SARS-CoV-2 variants: a model-based approach. Nat Commun. 2023 July 19;14(1):4325.

3. Hogan AB, Wu SL, Toor J, Mesa DO, Doohan P, Watson OJ, et al. Long-term vaccination strategies to mitigate the impact of SARS-CoV-2 transmission: A modelling study. PLOS Med. 2023 Nov 28;20(11):e1004195.

4. Australian COVID-19 Serosurveillance Network. Seroprevalence of SARS-CoV-2-specific antibodies among Australian blood donors: Round 4 update. 2023. Available from: https://www.kirby.unsw.edu.au/research/projects/serosurveillance-sars-cov-2-infection-inform-public-health-responses

5. GOV.UK. [cited 2025 Mar 7]. Winter Coronavirus (COVID-19) Infection Study: estimates of epidemiological characteristics, 15 February 2024. Available from: https://www.gov.uk/government/statistics/winter-coronavirus-covid-19-infection-study-estimates-of-epidemiological-characteristics-england-and-scotland-2023-to-2024/winter-coronavirus-covid-19-infection-study-estimates-of-epidemiological-characteristics-15-february-2024

6. Queensland Government Department of Health. Acute respiratory infection surveillance reporting. 2024 [cited 2024 Dec 11]. Available from: https://www.health.qld.gov.au/clinical-practice/guidelines-procedures/diseases-infection/surveillance/reports/flu

7. UK Health Security Agency. COVID-19 vaccine surveillance report: week 19. 2022. Available from: https://www.gov.uk/government/publications/covid-19-vaccine-weekly-surveillance-reports

8. Victorian Government Department of Health. Victorian COVID-19 surveillance report. State Government of Victoria, Australia; [cited 2024 Dec 11]. Available from: https://www.health.vic.gov.au/infectious-diseases/victorian-covid-19-surveillance-report

9. UK Health Security Agency. GOV.UK. [cited 2024 Dec 5]. National flu and COVID-19 surveillance report: 28 November (week 48). Available from: https://www.gov.uk/government/statistics/national-flu-and-covid-19-surveillance-reports-2024-to-2025-season/national-flu-and-covid-19-surveillance-report-28-november-week-48

10. Australian Government Department of Health and Aged Care. Australian Respiratory Surveillance Report 16–21 October 2024 to 17 November 2024. Australian Government Department of Health and Aged Care; 2024 [cited 2024 Dec 3]. Available from: https://www.health.gov.au/resources/publications/australian-respiratory-surveillance-report-16-21-october-2024-to-17-november-2024?language=en

11. UK Health Security Agency. GOV.UK. [cited 2024 Dec 12]. Winter Coronavirus (COVID-19) Infection Study: estimates of infection hospitalisation and fatality risk, 30 May 2024. Available from: https://www.gov.uk/government/statistics/winter-coronavirus-covid-19-infection-study-estimates-of-epidemiological-characteristics-england-and-scotland-2023-to-2024/winter-coronavirus-covid-19-infection-study-estimates-of-infection-hospitalisation-and-fatality-risk-30-may-2024

12. Ankit Rohatgi. WebPlotDigitizer. [cited 2024 Dec 19]. Available from: https://automeris.io

13. Hogan AB, Winskill P, Watson OJ, Walker PGT, Whittaker C, Baguelin M, et al. Within-country age-based prioritisation, global allocation, and public health impact of a vaccine against SARS-CoV-2: A mathematical modelling analysis. Vaccine. 2021 May 21;39(22):2995–3006.

14. Puhach O, Meyer B, Eckerle I. SARS-CoV-2 viral load and shedding kinetics. Nat Rev Microbiol. 2023 Mar;21(3):147–61.

S2. Additional results

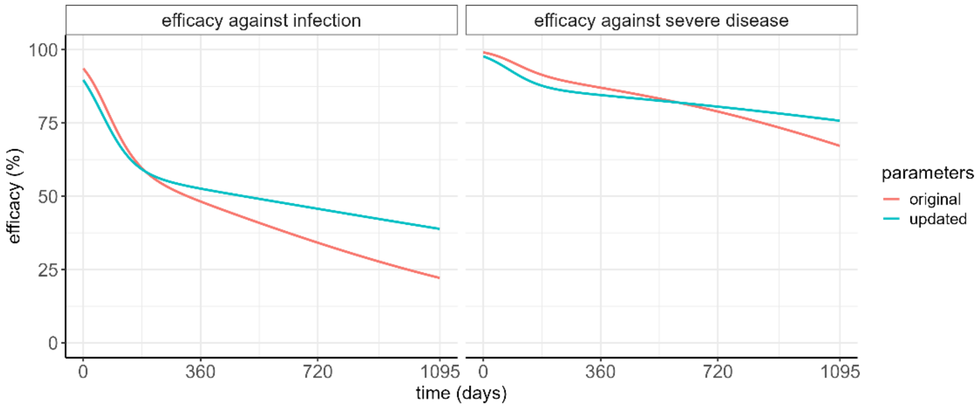

Figure S2. Illustration of varying the two parameters in the immunological model in order to produce efficacy curves with a more durable long-term immune response. Here, the red line ("original") illustrates the efficacy against infection (left hand panel) and severe disease (right hand panel) where we fix the shape parameter $k = 3.1$ and parameter corresponding to the long period of decay $h_l = 581$, as per the previous fitted immunological model. The blue line ("updated") illustrates efficacy against each outcome with the updated parameters that produce a more durable long-term immune response, where $k = 2.5$ and $h_l = 1000$. The "updated" parameters are those applied in the analysis in this study.

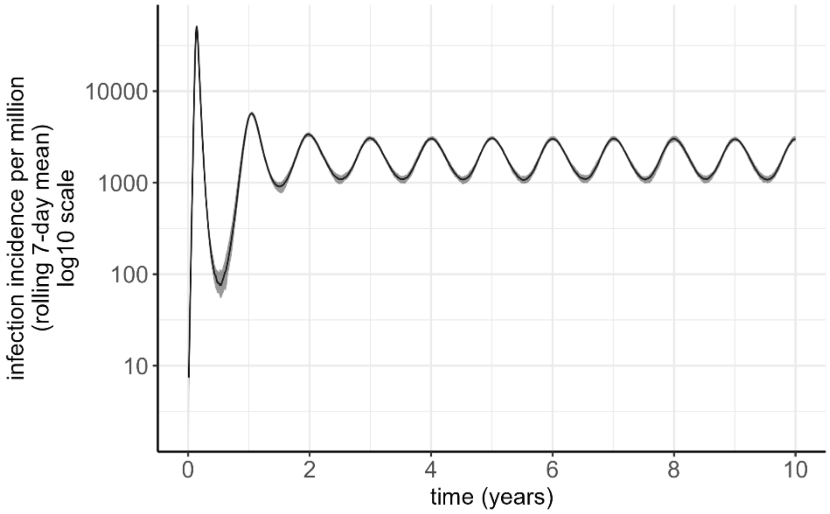

Figure S3. Infection incidence over time for a model simulation with no vaccination, and baseline parameters as specified in Table 1, illustrating infection endemicity reached within a 10-year burn-in period.

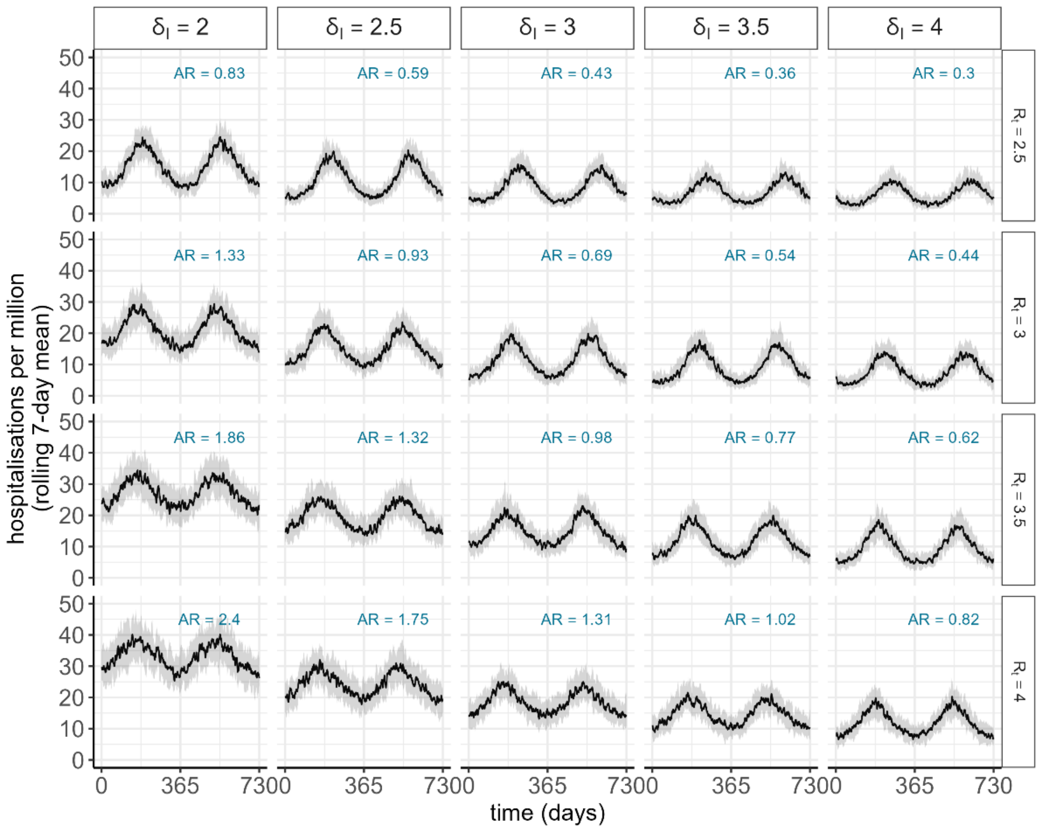

Figure S4. Model calibration without vaccination, across parameter sweeps varying the reproduction number R_0 and the level of immune recognition following infection δ_I. The black line shows the median trajectory, and the grey shaded region the 95% prediction interval generated from 20 model realisations. The attack rate (AR) is calculated as the total number of infections in a 12-month period divided by the total population.

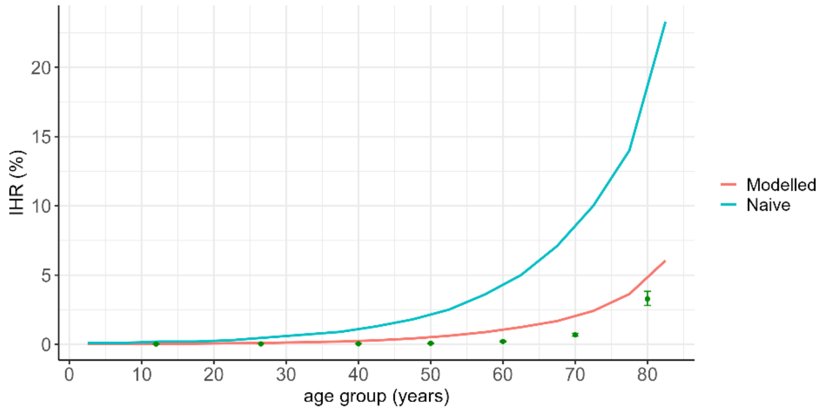

Figure S5. Infection hospitalisation ratio (IHR) (%) by age. The figure shows the model inputs (which represent the risk of hospitalisation with infection, for a "naïve" individual with no existing immunity), as the blue line, and the modelled outputs as the red line, which represent the proportion of modelled infections that result in hospitalisation for a population with immunity. The green points and bars depict recent estimates of the IHR for the UK, as published in the UK Health Security Agency "Winter Coronavirus (COVID-19) Infection Study: estimates of infection hospitalisation and fatality risk, 30 May 2024" (11)**.**

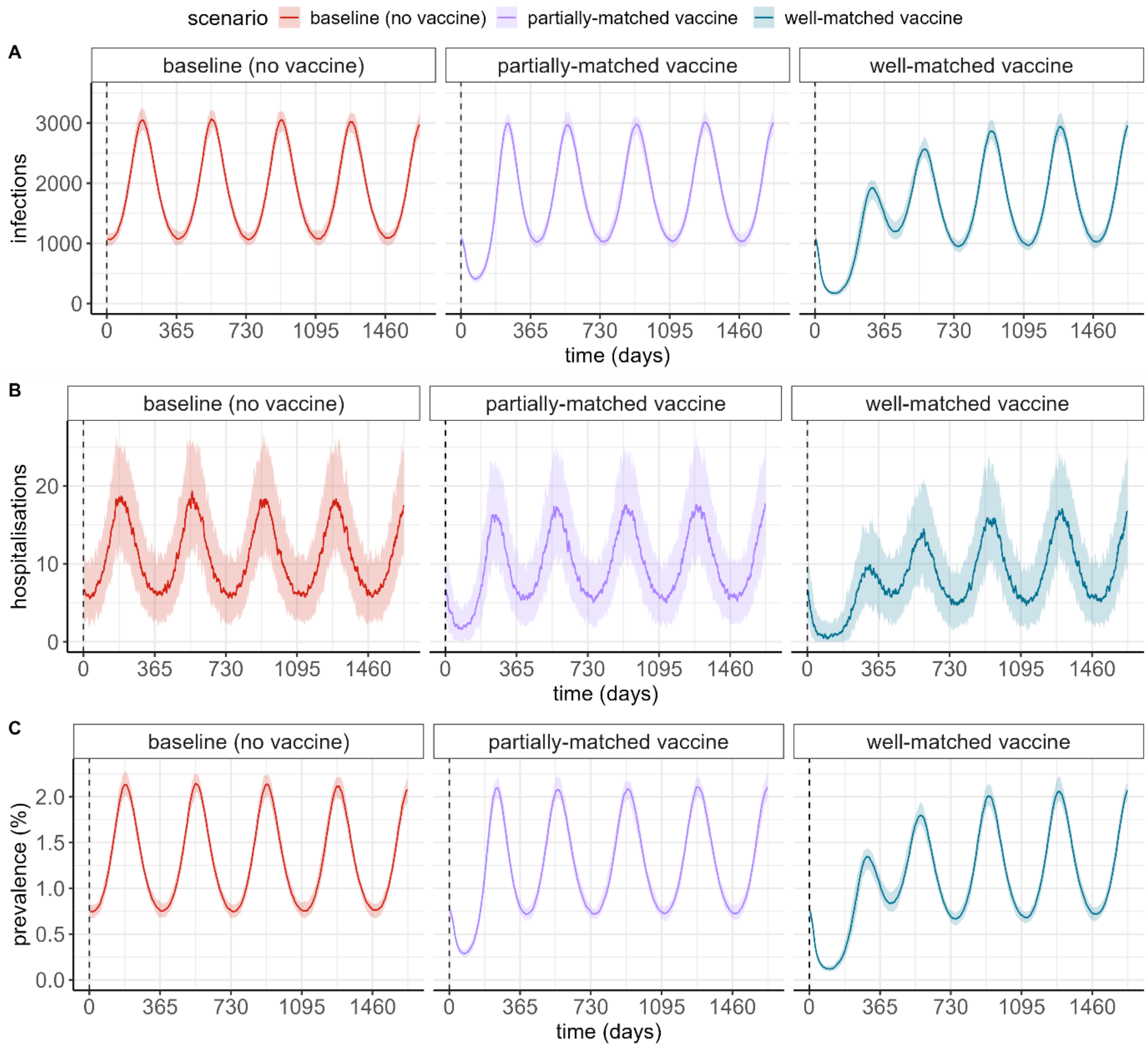

Figure S6. Daily infections (A), and daily hospitalisations (B), per million population, following a simulation burn-in period of 10 years, and in the absence of any new variant emergence. (C) Prevalence of SARS-CoV-2 infection. Note that the vertical dashed line in each panel represents the beginning of the simulated vaccine introduction, where a single vaccine dose is delivered to 80% of the population 15 years and older, over a 2-month period. Note that high vaccination coverage (80%) is used in these illustrative simulations, and that these simulations illustrate the 95% prediction intervals, generated from 20 model realisations, for the median trajectories shown in Figure 2.

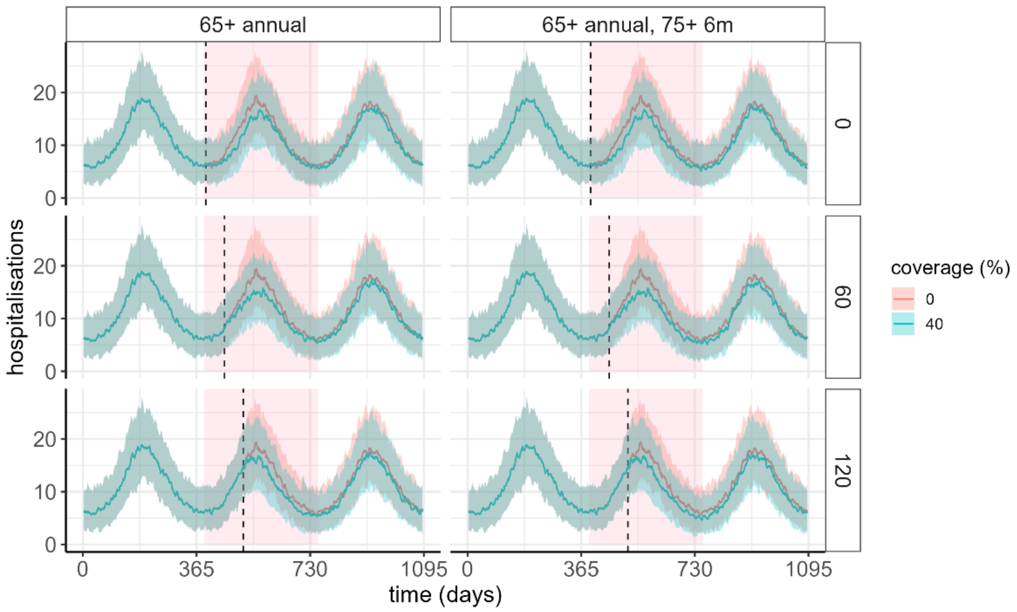

Figure S7. Vaccine impact shown as hospitalisations per million per day for a vaccine delivered at 40% coverage (blue line and shaded 95% prediction interval generated from 20 stochastic realisations), relative to a scenario with no vaccination (red line), for a scenario with no additional immune escape. The vertical dashed line indicates the time of vaccination commencement, at either 0, 60, or 120 days after the trough in infections (rows). The pink shaded region denoted the time window over which results are aggregated in the main text. The columns represent different vaccine strategies: either a single dose delivered to the 65+ population, or a single dose delivered to the 65+ population with an additional dose to the 75+ population after 6 months.

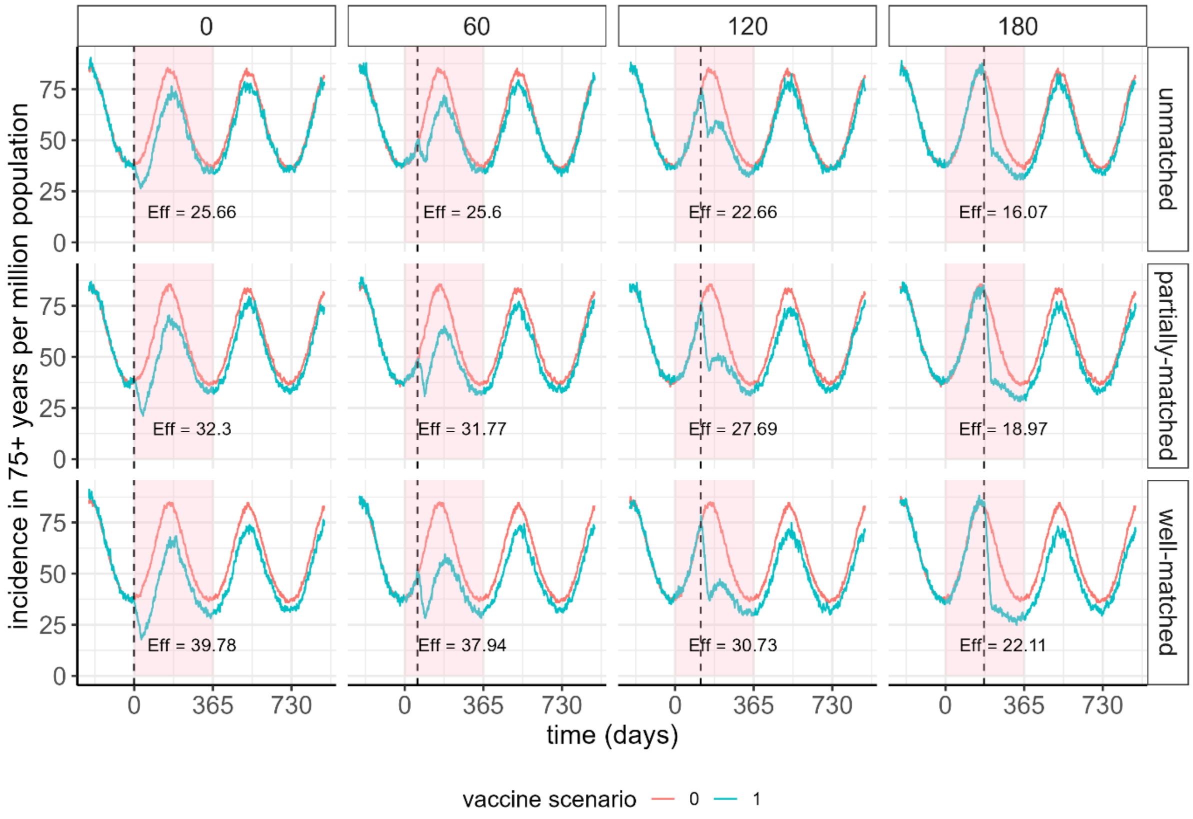

Figure S8. Modelled daily infections in the 75+ years age cohort, for a scenario with a vaccine delivered at high coverage (95%) in the 75+ years age cohort only (blue line), compared to a no-vaccination scenario. This is shown to estimate the individual-level benefit of vaccination at different timing relative to the COVID-19 epidemic (columns), and for different levels of vaccine matching to the circulating SARS-CoV-2 strain (rows). The calculated approximate vaccine effectiveness against hospitalisation over 12 months in the vaccinated cohort is annotated on each panel (%).

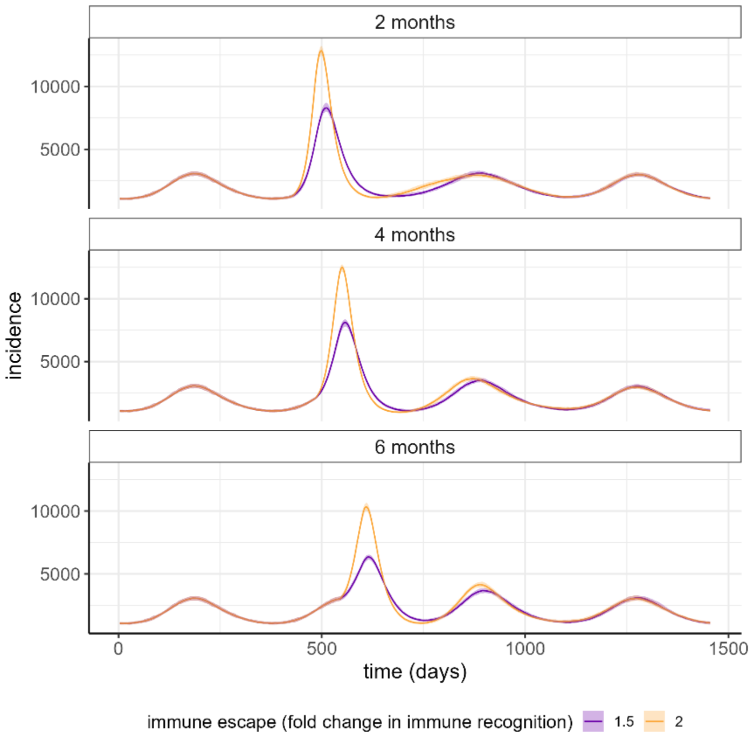

Figure S9. Simulated infections per day per million population, where an immune escape variant emerges either 2 months, 4 months, or 6 months following the seasonal trough (columns). Two levels of immune escape are simulated – a 1.5 (red) and 2 (blue) level fold drop in total immune recognition.

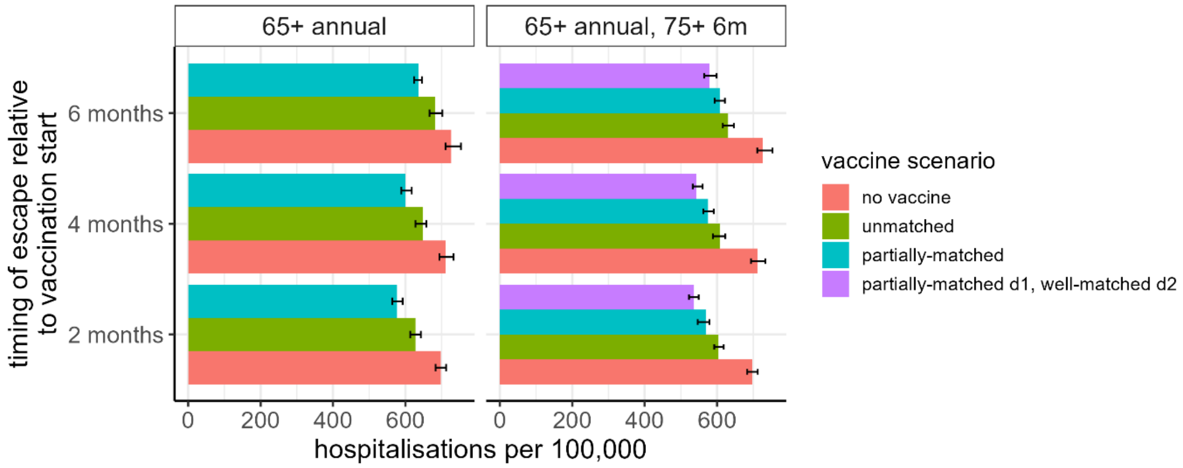

Figure S10. Total hospitalisations per 100,000 in the context of the emergence of a distinct variant strain either 2 months, 4 months, or 6 months following the seasonal trough (y-axis), for immune escape (a 1.5 level fold drop in immune recognition). Four vaccine scenarios are shown (coloured bars), for two vaccine delivery scenarios (columns): no vaccine (red); an unmatched vaccine (green), with $\delta = 1.5$, a partially-matched vaccine, with $\delta = 2$ (teal), and a partially matched first dose to the 65+ years population, with a second dose after 6 months delivered to the 75+ years population with a vaccine dose that is well-matched to the new variant strain (purple). The black bars represent 95% prediction intervals.

Table S3. Annual impact and averted events in terms of hospitalisations per 100,000 individuals, for routine vaccination scenarios. The modelled estimated baseline annual hospitalisations (i.e. in the no-vaccination group) was 414 (402–421) per 100,000. Values represent the median (95% prediction interval) across 20 stochastic model simulations.

Vaccine matching	Coverage	Relative timing	Schedule: 65+ annual			Schedule: 65+ annual, 75+ 6m		
			Hospitalisations per 100,000	Averted per 100,000	Proportion averted (%)	Hospitalisations per 100,000	Averted per 100,000	Proportion averted (%)
Unmatched	20%	0 days	399 (386, 408)	12 (-1, 28)	3 (-0.2, 6.6)	395 (388, 409)	15 (6, 31)	3.6 (1.3, 7.4)
		60 days	398 (385, 404)	15 (6, 29)	3.7 (1.6, 7)	397 (376, 406)	18 (0, 36)	4.4 (0, 8.7)
		120 days	398 (390, 408)	14 (0, 24)	3.3 (0.1, 5.8)	399 (389, 407)	15 (2, 26)	3.5 (0.5, 6.3)
	40%	0 days	382 (370, 393)	33 (13, 44)	7.9 (3.1, 10.6)	375 (364, 387)	34 (24, 51)	8.2 (6, 12.4)
		60 days	384 (374, 392)	31 (14, 38)	7.5 (3.6, 9.1)	382 (370, 392)	34 (14, 43)	8.2 (3.4, 10.3)
		120 days	389 (378, 399)	24 (11, 39)	5.9 (2.6, 9.3)	387 (377, 397)	24 (12, 40)	5.9 (3, 9.6)
	60%	0 days	374 (363, 382)	40 (22, 50)	9.7 (5.3, 12.2)	365 (350, 377)	48 (31, 62)	11.5 (7.8, 15)
		60 days	375 (361, 387)	42 (18, 52)	10.2 (4.5, 12.6)	367 (357, 379)	49 (25, 61)	11.7 (6.3, 14.6)
		120 days	378 (369, 386)	36 (25, 41)	8.6 (6.1, 9.9)	376 (368, 382)	38 (24, 44)	9.1 (6, 10.7)
Partially-matched	20%	0 days	395 (381, 407)	19 (3, 32)	4.6 (0.9, 7.8)	389 (380, 399)	25 (10, 32)	5.9 (2.6, 7.8)
		60 days	392 (385, 402)	18 (8, 29)	4.4 (2.1, 6.9)	390 (380, 398)	23 (6, 35)	5.5 (1.5, 8.4)
		120 days	397 (385, 408)	15 (1, 32)	3.7 (0.1, 7.6)	397 (386, 406)	16 (3, 31)	3.9 (0.7, 7.3)
	40%	0 days	376 (370, 383)	35 (22, 47)	8.5 (5.6, 11.1)	370 (358, 381)	41 (28, 59)	10.2 (7, 14.1)
		60 days	374 (367, 386)	38 (20, 52)	9.2 (5, 12.3)	370 (364, 384)	40 (19, 55)	9.7 (4.7, 13.1)
		120 days	384 (376, 393)	30 (13, 39)	7.2 (3.2, 9.3)	382 (372, 392)	34 (13, 40)	8.1 (3.3, 9.7)
	60%	0 days	358 (347, 371)	54 (40, 71)	13 (9.7, 16.9)	350 (342, 365)	61 (46, 76)	14.9 (11.2, 18.1)
		60 days	360 (347, 370)	55 (37, 67)	13.4 (9.1, 16.1)	353 (341, 364)	62 (40, 74)	14.9 (10, 17.7)
		120 days	371 (360, 380)	44 (30, 50)	10.5 (7.4, 12.1)	367 (358, 376)	47 (33, 55)	11.3 (8.1, 13.2)
Well-matched	20%	0 days	391 (377, 409)	20 (6, 38)	4.9 (1.5, 9.2)	384 (371, 403)	27 (11, 45)	6.4 (2.7, 10.7)
		60 days	391 (381, 400)	23 (9, 36)	5.4 (2.1, 8.5)	391 (379, 400)	24 (10, 31)	5.7 (2.5, 7.5)
		120 days	394 (383, 404)	20 (6, 29)	4.8 (1.6, 7)	394 (381, 406)	21 (7, 31)	4.9 (1.6, 7.5)
	40%	0 days	368 (355, 378)	44 (32, 59)	10.8 (7.9, 14.2)	361 (349, 369)	52 (42, 66)	12.6 (10.4, 16)
		60 days	370 (359, 379)	45 (23, 60)	10.8 (5.8, 14.3)	364 (355, 372)	49 (31, 64)	11.8 (7.8, 15.2)
		120 days	374 (364, 387)	39 (25, 47)	9.3 (6.2, 11.5)	373 (363, 386)	41 (25, 50)	10.1 (6.1, 12)
	60%	0 days	348 (340, 359)	65 (46, 80)	15.7 (11.5, 19)	335 (324, 345)	76 (63, 94)	18.4 (15.6, 22.5)
		60 days	348 (335, 361)	64 (44, 82)	15.6 (11, 19.5)	345 (329, 356)	70 (52, 88)	16.9 (12.9, 21)
		120 days	363 (354, 372)	51 (32, 62)	12.4 (7.9, 14.8)	360 (350, 368)	53 (35, 66)	12.7 (8.7, 15.8)

Table S4. Impact of different vaccine schedules and products in the context of the emergence of a new variant strain, for different scenarios for timing of immune escape relative to vaccination, assuming 60% coverage. Impact is shown as annual total and averted hospitalisations per 100,000 individuals. Values represent the median (95% prediction interval) across 20 stochastic model simulations.

Timing of immune escape	Schedule	Vaccine matching	Baseline hospitalisations per 100,000	Hospitalisations per 100,000	Averted per 100,000	Proportion averted (%)
2 months	65+ annual	Unmatched	697 (683, 713)	628 (613, 643)	71 (50, 90)	10.3 (7.3, 12.7)
		Partially-matched	697 (683, 713)	577 (564, 592)	122 (95, 142)	17.5 (13.9, 20.1)
	65+ annual, 75+ 6m	Unmatched	697 (683, 713)	604 (592, 618)	93 (80, 111)	13.3 (11.6, 15.6)
		Partially-matched	697 (683, 713)	570 (547, 579)	130 (117, 156)	18.7 (16.8, 22.2)
		Partially-matched d1, well-matched d2	697 (683, 713)	536 (522, 549)	160 (148, 179)	23.1 (21.3, 25.4)
4 months	65+ annual	Unmatched	712 (694, 733)	648 (628, 658)	69 (41, 95)	9.6 (5.9, 13.1)
		Partially-matched	712 (694, 733)	600 (589, 618)	110 (87, 137)	15.5 (12.5, 18.6)
	65+ annual, 75+ 6m	Unmatched	712 (694, 733)	609 (589, 623)	102 (83, 127)	14.5 (12, 17.7)
		Partially-matched	712 (694, 733)	576 (562, 591)	134 (109, 167)	18.8 (15.7, 22.7)
		Partially-matched d1, well-matched d2	712 (694, 733)	543 (533, 560)	165 (150, 185)	23.2 (21.4, 25.6)
6 months	65+ annual	Unmatched	726 (711, 754)	682 (667, 702)	44 (25, 78)	6 (3.4, 10.5)
		Partially-matched	726 (711, 754)	637 (624, 646)	92 (72, 116)	12.5 (10.1, 15.4)
	65+ annual, 75+ 6m	Unmatched	726 (711, 754)	630 (616, 647)	99 (70, 122)	13.7 (9.8, 16.3)
		Partially-matched	726 (711, 754)	608 (593, 622)	120 (99, 146)	16.5 (13.7, 19.4)
		Partially-matched d1, well-matched d2	726 (711, 754)	579 (564, 599)	151 (119, 174)	20.8 (16.7, 23.4)
